# Emendation of Appendix 9 of the International Code of Nomenclature of Prokaryotes to regulate the use of connecting vowels in compound names after stems ending in the same vowel

**DOI:** 10.1099/ijsem.0.006535

**Published:** 2024-10-01

**Authors:** Aharon Oren

**Affiliations:** 1 Institute of Life Sciences, The Hebrew University of Jerusalem, The Edmond J. Safra Campus, 9190401 Jerusalem, Israel

**Keywords:** connecting vowels, emendation, International Code of Nomenclature of Prokaryotes, International Committee on Systematics of Prokaryotes, orthography

## Abstract

Following a proposal to emend Appendix 9 of the International Code of Nomenclature of Prokaryotes with guidelines to regulate the use of connecting vowels in compound names after stems ending in the same vowel, I here report the outcome of the ballot on this proposal by the members of the International Committee on Systematics of Prokaryotes. The new guidelines to be incorporated in Appendix 9 are presented.

## Introduction

In December 2023, M.J. Pallen [1] published a proposal to emend Appendix 9 of the International Code of Nomenclature of Prokaryotes (ICNP) [2] with new guidelines to regulate the use of connecting vowels in compound names after stems ending in the same vowel. The original version of Appendix 9, section A(c) reads: **The connecting vowel is dropped when the following word element starts with a vowel.** The following emendation was proposed: **The connecting vowel is dropped when the preceding word element ends in the same vowel or when the following word element starts with a vowel**.

A public discussion of the proposal was held between 6 December 2023 and 5 June 2024 (see Article 13(b)(4) and Article 4(d) of the Statutes of the International Committee on Systematics of Prokaryotes (ICSP) [3]). The collated comments can be found in the supplementary information. In the course of this discussion, the following alternative emended version was proposed: **The connecting vowel is dropped when the following word element starts with a vowel. The connecting vowel may be dropped when the preceding word element ends in the same vowel**. M.J. Pallen endorsed the alternative emended version of his original proposed emendation.

The members of the ICSP voted on the proposals in June–July 2024. C.M. Manaia (Vice-Chair, ICSP) and H. Christensen (Vice-Chair of the ICSP Judicial Commission) collated the results of the vote. Out of the 46 members of the ICSP with voting rights, two endorsed the original emendation proposed by M.J. Pallen, 12 endorsed the alternative version proposed during the public discussion, five voted to retain the original version, and two abstained. Based on these votes, the following emendation of Appendix 9, section A(c) was accepted to provide guidelines for the use of connecting vowels in compound names:

**The connecting vowel is dropped when the following word element starts with a vowel. The connecting vowel may be dropped when the preceding word element ends in the same vowel**.

## Supplementary material

10.1099/ijsem.0.006535 Uncited Supplementary Material 1.

## Abbreviations

ICNP: International Code of Nomenclature of Prokaryotes
ICSP: International Committee on Systematics of Prokaryotes
