# Supplementary material for: Emendation of Appendix 9 of the International Code of Nomenclature of Prokaryotes to regulate the use of connecting vowels in compound names after stems ending in the same vowel
Source: Int J Syst Evol Microbiol. 2024 Oct 1;74(10):006535. doi: 10.1099/ijsem.0.006535 (PMC12453564; doi:10.1099/ijsem.0.006535)
Supplement: Uncited Supplementary Material 1. [file ijsem-74-06535-s001.pdf]

Comments posted on Slack

Collated and edited by Aharon Oren

10 January 2024
Aharon Oren: A paper entitled 'Use of connecting vowels after stems ending in the same vowel: a proposal to emend Appendix 9 of the International Code of Nomenclature of Prokaryotes' was published by Mark Pallen on 6 December 2023 in the IJSEM (vol. 73, article no. 006191; (https://doi.org/10.1099/ijsem.0.006191). This proposal to emend Appendix 9 is open for discussion until 5 June. ...

20 May 2024
Bernhard Schink, one of the IJSEM nomenclature reviewers, added the following comments about the proposal to emend Appendix 9 to change the use of connecting vowels in compound words: I have to admit that I can partly agree with Mark Pallen's arguments. The double -ii- words are hard to pronounce and there is no need to keep them; I do not see a case where there could be a loss of important information or of etymological accuracy by dropping the additional -i- in these cases. The situation is slightly different with the -oo- words of Greek origin. I doubt that the original word roots would be easily recognizable in Zogloea or chrococcus. I would therefore suggest to adopt the change by Mark Pallen but add a sentence that allows exceptions on a single-case basis.

20 May 2024
Marko Kostovski, one of the IJSEM nomenclature reviewers, added the following comments: I have read the article on modifying the way connecting vowels are used since it was published. This article was an inspiration to dig deeper into this topic, and although in the first place I did not like the idea, after consulting more articles on this topic, I personally think that this suggestion has its strengths. ... However, I like the elaboration on how the double "i" is confusing especially in the pronunciation. I also think that the elision of the additional -i would not make substantial difference in the etymology, as well as, what Bernhard proposed: "...add a sentence that allows exceptions on a single-case basis", but here I am not sure whether this could be proposed and voted as such. Considering the example given with alkali, I do not think that it should be used as a reference, but rather as an exception, as Aharon already mentioned.

In the following document:
The Form of Nominal Compounds in Latin - George D. Chase - Harvard Studies in Classical Philology, 1900, Vol. 11 (1900), pp. 61-72 - Department of the Classics, Harvard University
Stable URL: https://www.jstor.org/stable/310363 - there is is a short paragraph that is relation with this article, that I found useful:
-io, -ia stems in compounds end in a single i.
-io stems: anxi-fer, gaudi-vigens, laserpici-fer, medi-lunia, medi-terraneus, mendaci-loquus, negoti-nummius, offici-perda, sandali-gerula, ter/i-ceps.
-ia stems: glori-ficus, sapiendi-potens, sh'ri-cidium. This -i remains even before an initial vowel, as in anxi-animus, Medi-amna beside Medamna

Greek influence appears in the following: -io stems: Vergilio-cento, Vergilio-mastix, Claudio-polis, tertio-cerius, socio-fraudus, and with vowel weakening sociu-fraude (Plaut. Pseud. 362, cod. A), graju-gena.
-ia stems : vio-curus, Trojiu-gena, Maju-gena. Possibly vitu-/ero derives its first member from vitium and changes the i to u through the influence of the following labial.
The compounds of alius have the stem al'-, as ali'-quis, ali-quando, ali-ubi, ali-cubi, etc. The i that appears in these -io, -ia compounds does not arise from contraction or syncopation, but is the weak ablaut grade of the suffix -io. The -ia stems were treated exactly like the -io stems, so that we have really only one case to consider in compounds. The weak ablaut grade of -io had two forms -ī and -ĭ .

20 May 2024
Markus Göker: I wonder how you want to deal with names of Cyanobacteria validly published under the ICNafp. They are now also recognized as validly published under the ICNP. Therefore, they are within the reach of orthographic corrections, which according to the current Rule 61 can now be conducted by everybody everywhere unless they affect the first syllable. If -ii- in a cyanobacterial name gets corrected to -i- according to the ICNP and then corrected back to -ii- according to the ICNafp, of what use would that be for cyanobacterial nomenclature? As long as the ICNafp stipulates -ii- in such situations, there remains a problem. See ICNafp Article 60.10, Ex. 35, 36, 38.

21 May 2024
Aharon Oren: Do we always need to follow the use of connecting vowels as in classical Latin? Names and epithets such as Aquibacillus, Aquimonas, aquimarinus, and aquimaris would have sounded very strange, if not incorrect to Latin speakers in the Roman Empire. The genitive of aqua is aquae (1st declension) and not aqui (the genitive ending of the 2nd declension), so Aquaebacillus etc. would probably have sounded much better in classical Latin. We have a precedent in the epithet aquaeductus (of a conveyance of water), used for three genera. In this case, the authors used classical Latin, as the word aquaeductus (4th declension, genitive aquaeductus) is found in the dictionaries. If we had to form such a word from the component parts using the guidelines of Appendix 9, we would get aquiductus. In his 1993 book chapter, Thomas MacAdoo capitalized important words in the following sentence: “I must therefore remind the reader that BACTERIOLOGISTS ARE BOUND BY THE PROVISIONS OF THE CODE, without regard to other considerations.“ In view of the comments posted by Bernhard Schink and Marko Kostovski, I want to propose a slightly different version of Mark Pallen’s proposal.

The old version:
The connecting vowel is dropped when the following word element starts with a vowel.

Mark Pallen’s proposal:
The connecting vowel is dropped when the preceding word element ends in the same vowel or when the following word element starts with a vowel.

My alternative proposal that we can present in the ballot:

The connecting vowel is dropped when the following word element starts with a vowel. The connecting vowel may be dropped when the preceding word element ends in the same vowel.

21 May 2024
Markus Göker: I would welcome this change. However, I wonder whether the ambiguity it creates might lead to attempts at orthographic corrections, which under the current Rule 61 can now be made by anyone anywhere, provided they do not affect the first syllable. In particular, there may be attempts to standardize the spelling of compound words with the same left or right component in terms of the use of the connecting vowel.What about: **The connecting vowel is dropped when the following word element starts with a vowel. The connecting vowel may be dropped when the preceding word element ends in the same vowel. In case of doubt, the original spelling is preferred.** We must also bear in mind that there are already many words with -ii-, caused by a connecting vowel -i-, and I doubt that we want them all to be orthographically corrected.

21 May 2024
Aharon Oren: Markus, thanks for these comments. However, it is not clear what it meant with 'the original spelling'. If I understand well, the change proposed by Mark Pallen is for new names and is not intended for retroactive correction of existing names.

21 May 2024
Markus Göker: This may indeed have been his intention. However, Appendix 9 can be consulted by anyone who wishes to determine whether names should be corrected, long after the names have been proposed. The term "original spelling" is used in my proposal as in Rule 61. If we include "The connecting vowel may be dropped when the preceding word element ends in the same vowel", one author may conclude that in a particular case the connecting vowel should have been dropped, but wasn't, and correct the name. Another author might conclude that in a particular case the connecting vowel shouldn't have been dropped, but was, and correct the name. This is of particular interest when some usages in classical Latin or in previously published taxon names suggest that the connecting vowel should be dropped, and other usages suggest that it should be retained.

23 May 2024
Stefano Ventura, one of the IJSEM nomenclature reviewers, added the following comments: I have to say that I was not able to find a affirmed use of the double ii in Latin. I confirm that the connecting i took a prominent role early in the development of Latin but at the same time many exceptions existed. We cannot force microbiologists to study Latin morphology up to be able to determine if a connecting i is to be correctly applied or not. In addition, this apply to Latin words, but I am not sure we can use the same criteria when Neo Latin words are created.
So my suggestion is to keep the simple request to add a connecting i when making compound words. For the special case of the double ii, I agree with Bernhard, but would extend the possibility to even skip the use of connecting i in single cases, but only if a valuable explanation is given that relies on classical Latin forms.

24 May 2024
Mark Pallen: Thanks for all this helpful discussion and scholarship. I am happy with Aharon's suggestion that we vote on this form of words: **The connecting vowel is dropped when the following word element starts with a vowel. The connecting vowel may be dropped when the preceding word element ends in the same vowel.**

24 May 2024
Markus Göker: Thanks for your comment. Would also be fine with: **The connecting vowel is dropped when the following word element starts with a vowel. The connecting vowel may be dropped when the preceding word element ends in the same vowel. In case of doubt, the original spelling is preferred.** As discussed, this does not change the picture when proposing new names, but it may help against unwarranted corrections.

24 May 2024
Mark Pallen: I would also be happy for the voting to begin on June 4th. ...

3 June 2024
Mark Pallen: I think this overcomplicates things. If someone wants to correct things retroactively, saying "in case of doubt such and such option is preferred" will not force them to stop. In my opinion there is no doubt that *Posidonimonas* is preferable to *Posidoniimonas*, so who decides that "in case of doubt" applies? It is only inertia that would stop me retroactively changing all such cases, if the rules allow it. (edited)

3 June 2024
Markus Göker: Of course, orthographic corrections can be made anyway, but the inclusion of a phrase like "in case of doubt the original spelling is preferred" would tell others whether or not they have to accept the correction. By including the "may" in "**The connecting vowel may be dropped when the preceding word element ends in the same vowel**" the action is made optional, which is likely to generate doubt. Not only subjective doubt, but objective doubt, as the wording does not result in certainty. The resulting objective doubt would get removed again by including "in case of doubt the original spelling is preferred". In general, more detailed regulations may be easier to apply in practice since they remove ambiguity that would remain if regulations were simpler.

3 June 2024
Markus Göker: The underlying issue is whether anybody has an interest in retroactive changes of spellings because of -i- vs, -ii- or whether this is supposed to only apply to new names.

3 June 2024
Aharon Oren: In my opinion the longer version does not clarify anything, it only will cause confusion. My version ("version 2") simply proposed that both options are allowed. The question is now, whether we should present both version 2 and version 3 in the ballot or (my

proposal) only version 2. We still have two days to decide. If we do not have a consensus, I will present both versions in the ballot and let the members decide.

3 June 2024
Markus Göker: Well, if it becomes just "**The connecting vowel may be dropped when the preceding word element ends in the same vowel",** how should databases such as LPSN decide which version to choose, the -i- one or the -ii- one? The addition I've proposed would clarify which version to select in case of an already validly published name, without the need to give this to the JC.

3 June 2024
Aharon Oren: Simply use the version used by the authors in IJSEM papers, the nomenclature reviewers no longer should to suggest corrections, and the list editors do not have to correct effectively published names because the use of -i- or -ii=. In my opinion, everything will be simple, also for databases. Why should names that were already validly published be changed retroactively? This is not a grammatical issue such as the gender of adjectival epithets that must follow the gender of the genus name (at least for prokaryotes, plants and animals, some virus taxonomists may have different ideas ...).

3 June 2024
Markus Goker: The point is that "use the version used by the authors in IJSEM papers" is not specified in your version of the text. I suggested that this be specified. As for "Why should names that were already validly published be changed retroactively?", according to the 2022 revision of the ICNP, this can be done in all cases of grammatical or orthographic corrections by anyone, anywhere. Given this situation, precautions may be warranted. The same applies to names proposed after the proposed change to the ICNP we are discussing is ratified. If authors start using -i- instead of -ii-, others may simply switch to -ii- because they think it is better -- and have the right to make orthographic corrections.Conversely, I wonder what the downside would be of adding "In case of doubt, the original spelling is preferred". It does not seem to harm anyone, unless we consider it a significant burden to have to read an extra sentence. However, the sentence would remove ambiguity and may well prevent the proliferation of different spellings of the same name.

3 June 2024
Aharon Oren: Corrections are only needed when errors were made in the past. If both forms are considered acceptable, there is nothing to correct, also not when an author is in favor of one version. In my opinion, 'in case of doubt ..." is confusing,

3 June 2024
Markus Göker: We still have rules like Rule 58 "If doubt exists about different spellings of the same name or epithet, or if two spellings are sufficiently alike so as to be confused, the question should be referred to the Judicial Commission, which may issue an Opinion. If one of the spellings is preferred by the Judicial Commission, that spelling should be used by succeeding authors." The point is that "may be dropped" causes ambiguity: does the connecting vowel

need to be dropped in a particular case or not? And then the issue arises that I have mentioned earlier: if the connecting vowel has been dropped after a certain word component in one to many names, does it always need to be dropped after that word component? Consistency is an argument here and elsewhere. Or, alternatively: if the connecting vowel has been dropped before a certain word component in one to many names, does it always need to be dropped before that word component? Conversely, I wonder what is confusing in "In case of doubt, the original spelling is preferred." For instance, is the sentence supposed to be ambiguous? I think "preferred" is clear, "original spelling" is already defined in the ICNP, and "in case of doubt" would refer to the previous sentence.
